# Interpreting the results of a modified gravity model: examining access to primary health care physicians in five Canadian provinces and territories

**DOI:** 10.1186/1472-6963-12-230

**Published:** 2012-08-01

**Authors:** Valorie A Crooks, Nadine Schuurman

**Affiliations:** 1 Department of Geography, Simon Fraser University, 8888 University Dr, Burnaby, BC, V5A 1S6, Canada

## Abstract

**Background:**

Primary health care (PHC) encompasses an array of health and social services that focus on preventative, diagnostic, and basic care measures to maintain wellbeing and address illnesses. In Canada, PHC involves the provision of first-contact health care services by providers such as family physicians and general practitioners – collectively referred as PHC physicians here. Ensuring access is a key requirement of effective PHC delivery. This is because having access to PHC has been shown to positively impact a number of health outcomes.

**Methods:**

We build on recent innovations in measuring potential spatial access to PHC physicians using geographic information systems (GIS) by running and then interpreting the findings of a modified gravity model. Elsewhere we have introduced the protocol for this model. In this article we run it for five selected Canadian provinces and territories. Our objectives are to present the results of the modified gravity model in order to: (1) understand how potential spatial access to PHC physicians can be interpreted in these Canadian jurisdictions, and (2) provide guidance regarding how findings of the modified gravity model should be interpreted in other analyses.

**Results:**

Regarding the first objective, two distinct spatial patterns emerge regarding potential spatial access to PHC physicians in the five selected Canadian provinces: (1) a clear north–south pattern, where southern areas have greater potential spatial access than northern areas; and (2) while gradients of potential spatial access exist in and around urban areas, access outside of densely-to-moderately populated areas is fairly binary. Regarding the second objective, we identify three principles that others can use to interpret the findings of the modified gravity model when used in other research contexts.

**Conclusions:**

Future applications of the modified gravity model are needed in order to refine the recommendations we provide on interpreting its results. It is important that studies are undertaken that can help administrators, policy-makers, researchers, and others with characterizing the state of access to PHC, including potential spatial access. We encourage further research to be done using GIS in order to offer new, spatial perspectives on issues of access to health services given the increased recognition that the place-based nature of health services can benefit from the use of the capabilities of GIS to enhance the role that visualization plays in decision-making.

## Background

Broadly speaking, primary health care (PHC) encompasses an array of health and social services that focus on preventative, diagnostic, and basic care measures to maintain wellbeing and address illnesses before they become severe and/or life threatening [1]. In Canada, PHC refers to the provision of first-contact health care services by providers such as family physicians and general practitioners – collectively referred to throughout this article as PHC physicians [2]. Despite the importance of having access to PHC physicians, little is known about how similar or different PHC delivery is between provincial jurisdictions Canada [3]. This is troubling given that Canadian studies have repeatedly demonstrated problems with equity in access to health care for specific places and groups [4-6]. As a result, lack of service availability and needs-based barriers, such as proximity to services, prohibit some citizens from having reasonable access to PHC [7]. This can be particularly so in rural and remote areas, where service delivery must typically be more sensitive to issues of distance minimization rather than even distribution [8]. In other words, enabling good spatial access to PHC services in rural and remote areas can be challenging due to the often great distances between these communities and also to major urban centres.

In a systematic review of the role of PHC within health systems, Kringos et al. [9] identified access as a key requirement of effective PHC delivery. PHC is uniquely positioned with regard to enabling access, as it is both the most spatially and financially accessible form of health care when compared with other health service tiers that rely upon specialists and technicians [10]. Having access to PHC has been shown to positively impact health outcomes in a variety of ways. For example, analyses focused on PHC physician-to-population ratios have shown a positive correlation between physician numbers and self reported health indicators [11] and life expectancy [12], and a negative correlation between physician numbers and colorectal cancer [13] and mortality from both heart disease and cancers [14]. Enabling access to PHC physicians is thus valuable to individuals and also health care systems.

Ensuring citizens’ access to health care services is fundamental to delivering PHC [15-17]. There are a number of ways to conceptualize access to PHC, ranging across factors as diverse as having the capacity to pay for care [18], to the operating hours of clinics [11], or how easily a person can obtain needed care in a time that is appropriate to the urgency of the health problem [19]. Central to each of these conceptualizations is that people require spatial access to PHC, whereby they must be able to *get to* the service in a timely manner. Delivering PHC in a spatially accessible way increases the equity (i.e., fairness) with which care is delivered [7,20,21].

Access to health services in general is conceptualized in a number of ways. In the broadest sense, there is potential spatial access (e.g., having a clinic close by that one can visit), potential aspatial access (e.g., having culturally appropriate care available that one can access), realized spatial access (e.g., actually going to a close by clinic), and realized aspatial access (e.g., seeing a doctor from one’s own cultural group) [22,23]. In this article we focus specifically on potential spatial access to PHC physicians. By spatial we mean that we are considering *geographic proximity* to be indicative of access, versus access models that do not consider this form of access, and by potential we mean that we are considering the *possibility* of service use as an expression of access, versus access models that incorporate actual service use. We use potential spatial access as our focus as there is no reliable or comparable utilization data that is readily available for cross-province analysis. Potential spatial access is determined primarily by the distance between patient and physician and the availability of care relative to local demand [24]. Traditional ways of measuring potential spatial access have focused on fairly simplistic measures of factors such as average travel time to a physician’s clinic, average travel distance to a physician’s clinic, and facility counts within geographically bounded units [25-29]. More recently, geographic information systems (GIS) have been used to evaluate potential spatial access to PHC. GIS analyses have enabled the inclusion of new variables into studies of health service access, such as patient catchments and road network travel time, while allowing the simultaneous consideration of more variables than can typically be included in statistical analyses without spatial components. Such variables allow for gaps and overlaps in service availability to be clearly identified [30,31].

**Figure 1:**
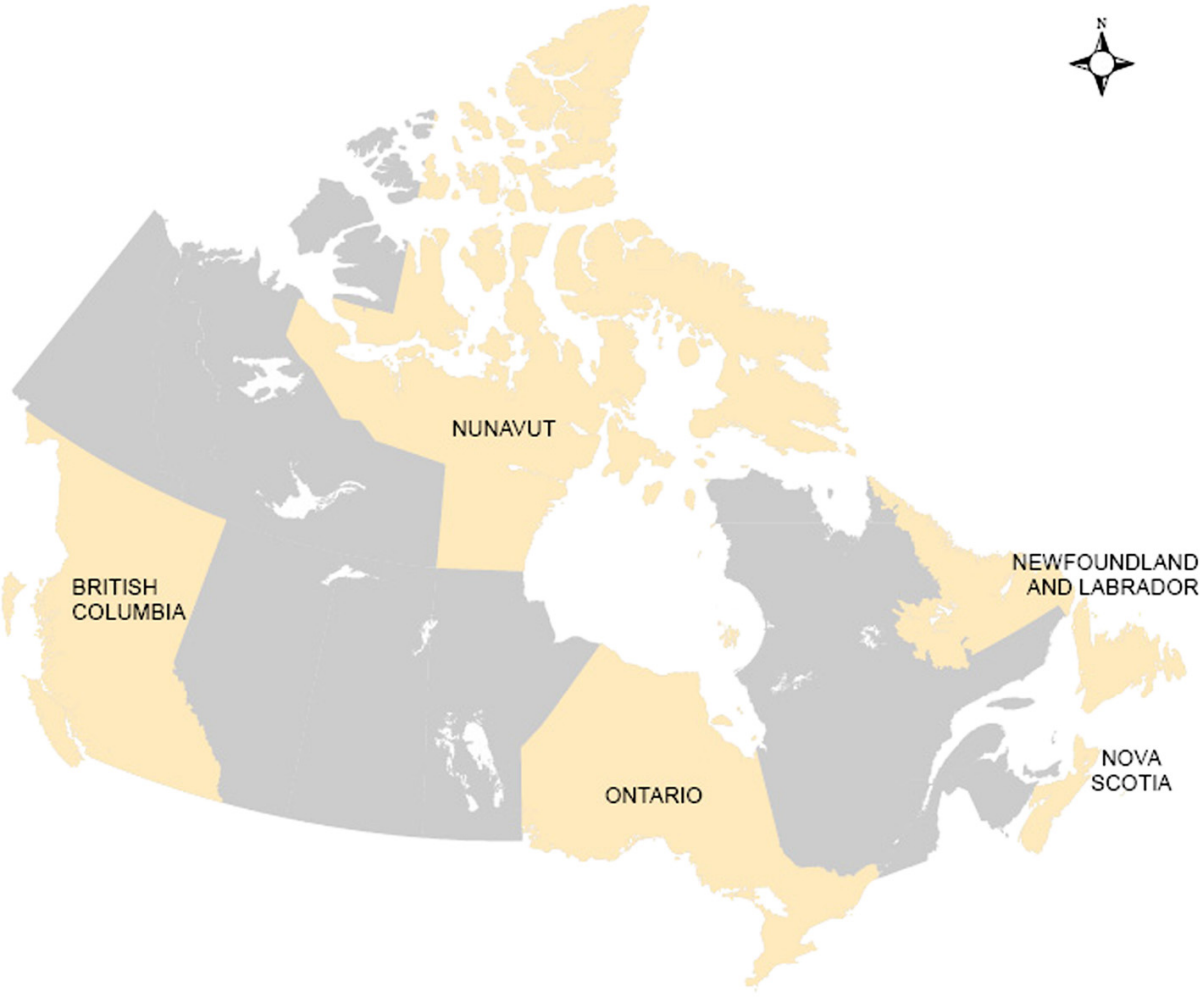
** Provinces and territories of focus.** This map displays the five provinces and territories that are examined in the present article.

In this article we build on recent innovations in measuring potential spatial access to PHC physicians using GIS by running and then interpreting the findings of a *modified* gravity model. Most broadly, gravity models examine flows or movements between two sites, such as between patients’ residential locations and a doctor’s office. The gravity model equation is based on Newton’s Law of Gravitation. The gravity model is thought to be the most reliable method of measuring spatial access because it takes into account decreasing likelihood of access with increased distance from service sites [32], and has been used in other studies of potential spatial access to health care [24,33]. In this analysis, we apply a gravity model that incorporates road network travel time, this being the modification, to access to PHC physicians. Road network travel time is a *theoretical* representation of real-world drive time. In a previous article we introduced the model employed in the present analysis and compared it to the kernel density model (where the kernel density model estimates a smooth probability density using multivariate or univariate data) [34]. Arguing that little attempt has been made to determine how to most accurately depict potential spatial access to PHC physicians, we tested these two models using data for the Canadian province of Nova Scotia. We concluded in the previous article that the modified gravity model is superior to the kernel density model as it incorporates road network travel time, versus crow-fly distances, and uses real census block population counts, which avoids the issue of including non-populated areas (e.g., parks, lakes) in the analysis [34]. Now that the model has been introduced and tested, in the present article we run it for five Canadian provinces and territories depicted in Figure 1. These provinces and territories were purposefully selected for their differences in population density and topography. Our objectives are to present the results of the modified gravity model in order to: (1) understand how potential spatial access to PHC physicians can be interpreted in these Canadian jurisdictions, and (2) provide guidance regarding how findings of the modified gravity model should be interpreted in other analyses so that future users of this model can look to the current article for insight into how to approach this process. 

## Methods

We first outline the data sources for this analysis and then provide an overview of the modified gravity model protocol that has been published in full elsewhere [34].

### Summarizing the modified gravity model

The modified gravity model is:

$$A_{i}=\sum_{j}\frac{S_{j}}{D_{j}f(t_{\mathrm{ij}})}$$

A_i_ is the access measure for a particular dissemination block, block i. If a study area is subdivided into 100 dissemination blocks, then the value of i ranges from 1 to 100. The access measure for one dissemination block is equal to the sum of the access values for each accessible physician location. The accessibility of a particular physician location depends on three main variables: supply (S_j_), demand (D_j_) and travel time (t_ij_), where t_ij_ represents the travel time from dissemination block i to physician location j. The variable j identifies a particular physician location and, in the summation above, ranges from 1 to however many physician locations are accessible to dissemination block i. In our study, we considered physician locations accessible to a dissemination block if they were within a 2 hour road travel time. If there are no physician locations within 2 hours of a dissemination block, it has an access of 0. This is the minimum value of A_i_.

In our modified gravity model, supply was considered to be the number of physicians at a physician location. For demand, we did not use actual usage statistics. Demand at each physician location was calculated prior to calculating dissemination block access scores using the following formula:

$$D_{j}=\sum_{k}\frac{P_{k}}{f(t_{\mathrm{kj}})}$$

The demand at a physician location is equal to the sum of the dissemination block populations within 2 hours, with each population weighted by a travel time impedance function. In the summation, the variable k ranges from 1 to however many population blocks are within 2 hours of physician location j. t_kj_ is the travel time from dissemination block k to physician location j. f(t) is a travel time impedance function; f(t_kj_) is the travel time impedance between dissemination block k and physician location j. It is the same function used in the access calculation and is defined as follows:

$$\begin{array}{l} f(t_{\mathrm{ij}})=1\;\text{for}\;t_{\mathrm{ij}}\leq 10\text{minutes}\\ f(t_{\mathrm{ij}})=\frac{t_{\mathrm{ij}}}{10}\;\text{for}10\leq t_{\mathrm{ij}}\leq 120\text{minutes}\\ f(t_{\mathrm{ij}})=\infty \;\text{for}\;t_{\mathrm{ij}}>120\;\text{minutes(no access: }\frac{1}{\infty }\text{=}0) \end{array}$$

This impedance function is similar to one used by McGrail and Humphreys [35]. Dissemination blocks within 10 minutes travel time of a PHC physician were assigned no travel impedance: *f*(*t*_*ij*_) = 1. Those farther than 120 minutes from a physician were considered to have no access (infinite travel impedance). The travel impedance between 10 and 120 minutes increases linearly (ranging from 1 at 10 minutes to 12 at 120 minutes) so that a dissemination block 80 minutes from a PHC physician has 4 times the travel impedance of one 20 minutes away. Clearly, individuals may vary in the extent to which adding 50% to 100% travel time affects their likeliness to pursue primary health care. This is a persistent issue that we resolved by using a linear gradient [36].

For all travel times, the value of the impedance function increases monotonically with travel time. Hence, a dissemination block which is further from a physician location imposes a lower demand, *P*_*k*_*/f*(*t*_*kj*_), than a closer dissemination block with the same population size. Similarly, a physician location which is further from a dissemination block has a lower access value, *S*_*j*_*/*(*D*_*j*_*f*(*t*_*ij*_)), than a closer one.

In this analysis, we used a 120 minute maximum for assessing potential spatial access. There are no standard drive time precedents for potential spatial access to PHC physicians in Canada. Because of this, we had to subjectively determine and justify a drive time maximum. We opted to use a 120 minute maximum as we believe this reflects what Canadians in rural communities, and in some remote regions as well, will deem to be reasonable for a one-way drive time to a PHC physician. There are varying cut-offs for reasonable access in the literature; however, all are subjective [37,38]. In this case, we followed the BC Ministries of Health Service guidelines for acute patient care [39]. As we note later in the article, there are certainly a number of limitations associated with our use of the 120 minute maximum drive time.

To calculate road travel times, dissemination blocks were paired with *each* PHC physician within 120 minutes of travel time. Road travel times for each dissemination block-PHC physician pair were calculated using an Origin Destination Cost Matrix (ODCM) tool. This tool is part of the Network Analyst extension in ESRI’s ArcMap 9.3. Dissemination block centroids within 2500 meters of a road and with a population density greater than 1 person per 5 square kilometers were used as origins, and physician postal code centroids within 2500 meters of a road were input as destinations.

Using the dissemination block-PHC physician pair travel time values in the resulting matrix, the travel time impedance function, *f*(*t*_*ij*_), was calculated for each pair. This value was added as a column to the matrix. In each ODCM row, the destination physician postal code and origin dissemination block were identifiable by unique identifiers (unique identifying sequences of letters and numbers for each dissemination block and postal code). These unique identifiers allowed us to perform table joins between the ODCM (which is a table) and the physician and dissemination block attribute tables. These joins were temporary joins performed with SQL queries in Microsoft Access in order to calculate demand values at each physician location and access values for each dissemination block. A column for demand values was added to the PHC physician location attribute table. An SQL query, which joined the ODCM, physician location table and dissemination block table and performed the necessary summation, was executed in order to calculate the demand values. These demand values were then used to determine access scores for each dissemination block. An access score column was added to the dissemination block attribute table and an SQL query was run to calculate these scores. Again, this query involved joining the ODCM, physician location table and dissemination block table and performing a summation.

### Data

PHC physicians: To conduct this analysis, we purchased 12 months of access to the MDSelect Canadian Medical Directory in 2008–09. This is a comprehensive, regularly-updated address directory of Canadian medical professionals that can be searched by specialty. The directory is only available through private purchase, though there are no restrictions on who can purchase access to it. Addresses of PHC physicians practicing in the five targeted Canadian provinces and territories were obtained from data tables. We used only data for physicians with either a ‘General Practice’ or ‘Family Medicine’ designation in the directory as both groups are considered PHC physicians within the Canadian system [34]. Geo-coding was based on postal codes rather than street addresses as some physicians were listed by rural post office box only. DMTI Platinum Suite 2008 postal codes were obtained from Canada Census for geo-coding.

Population size: We used Statistics Canada 2006 census block centroids (point data) to represent population size. Demographic information and other calculations were referenced to dissemination areas or blocks; these are the smallest areas for which all demographic variables are publicly reported by Statistics Canada. These data are available for academic researchers in Canada.

Road travel time: Road travel times were calculated using the DMTI 2008 CanMap RouteLogistics roads dataset for each of the provinces and territories. The DMTI road data includes detailed information for each road segment including distance, speed limits, and travel direction (e.g., one-way or two-way streets). These road data are available to university researchers through a data-sharing agreement with Statistics Canada. They are available to academic researchers across Canada.

## Results

**Figure 2:**
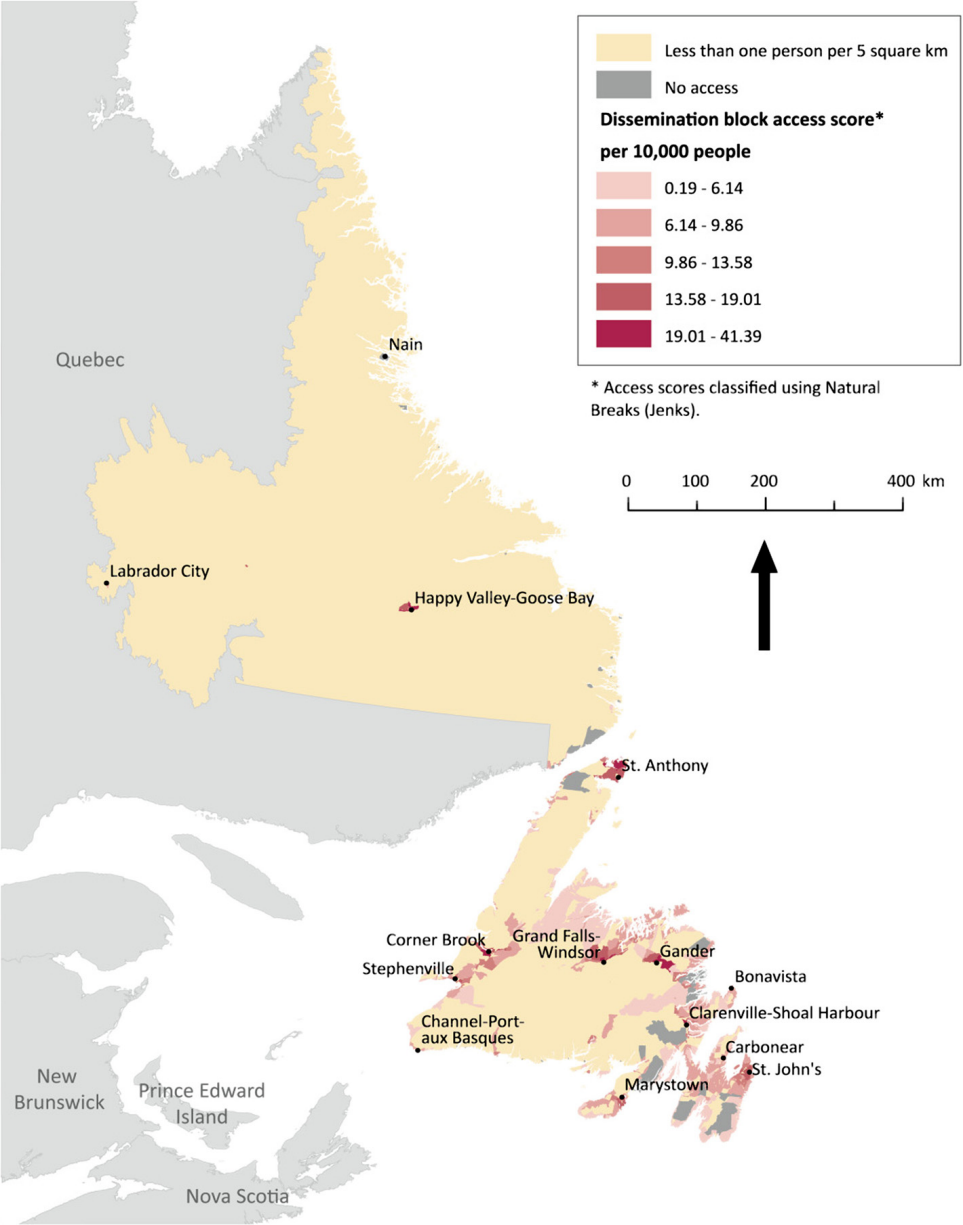
** Potential spatial primary health care physician access in Newfoundland and Labrador.** Potential spatial access to primary health care physicians in Newfoundland and Labrador. Whilst the most populated areas enjoy varying degrees of access, remote areas are generally lacking services.

**Figure 3:**
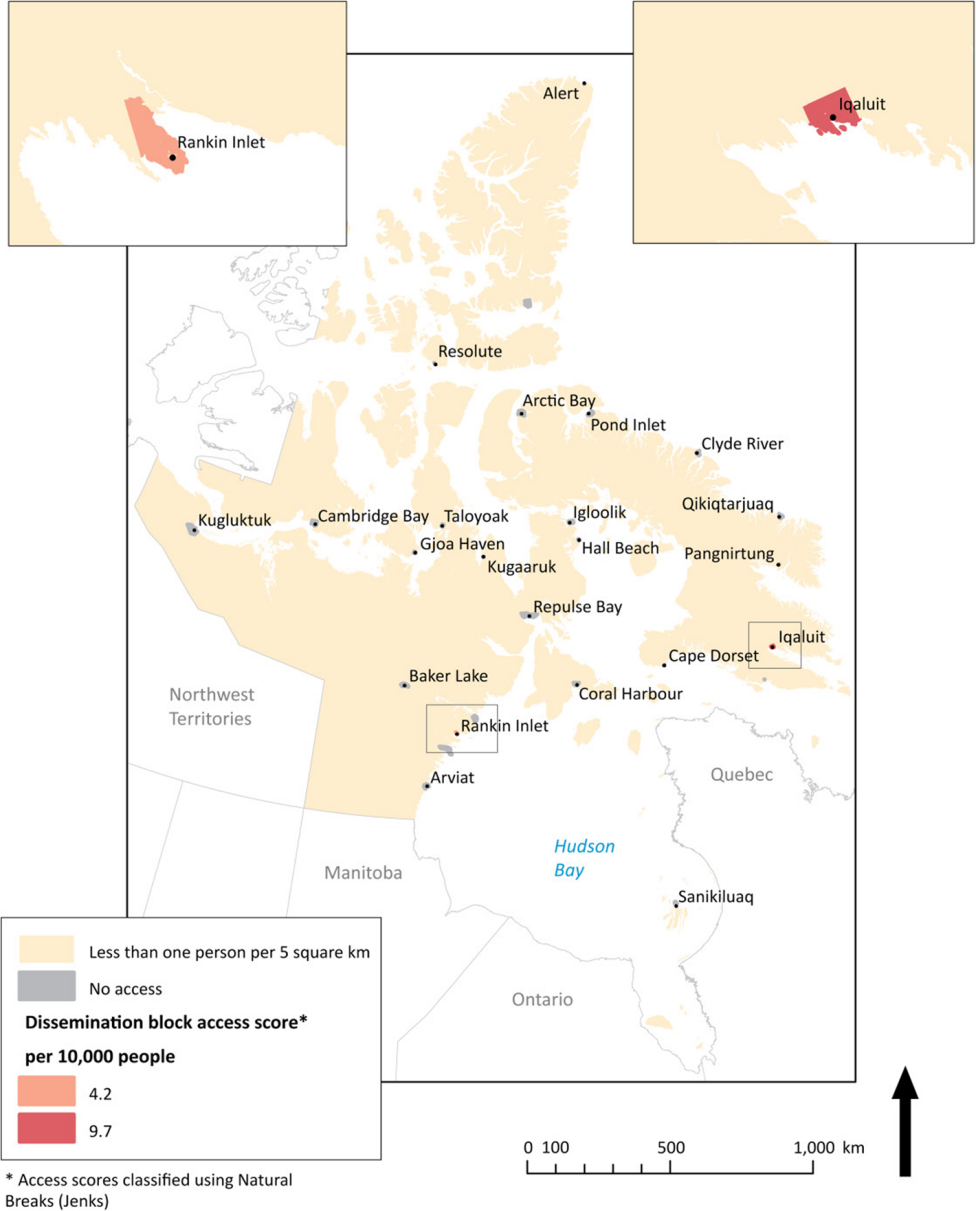
** Potential spatial primary health care physician access in Nunavut.** Potential spatial access to primary health care physicians in Nunavut. Here access is excellent within the two most populated cities and missing entirely outside the borders where the roads end.

**Figure 4:**
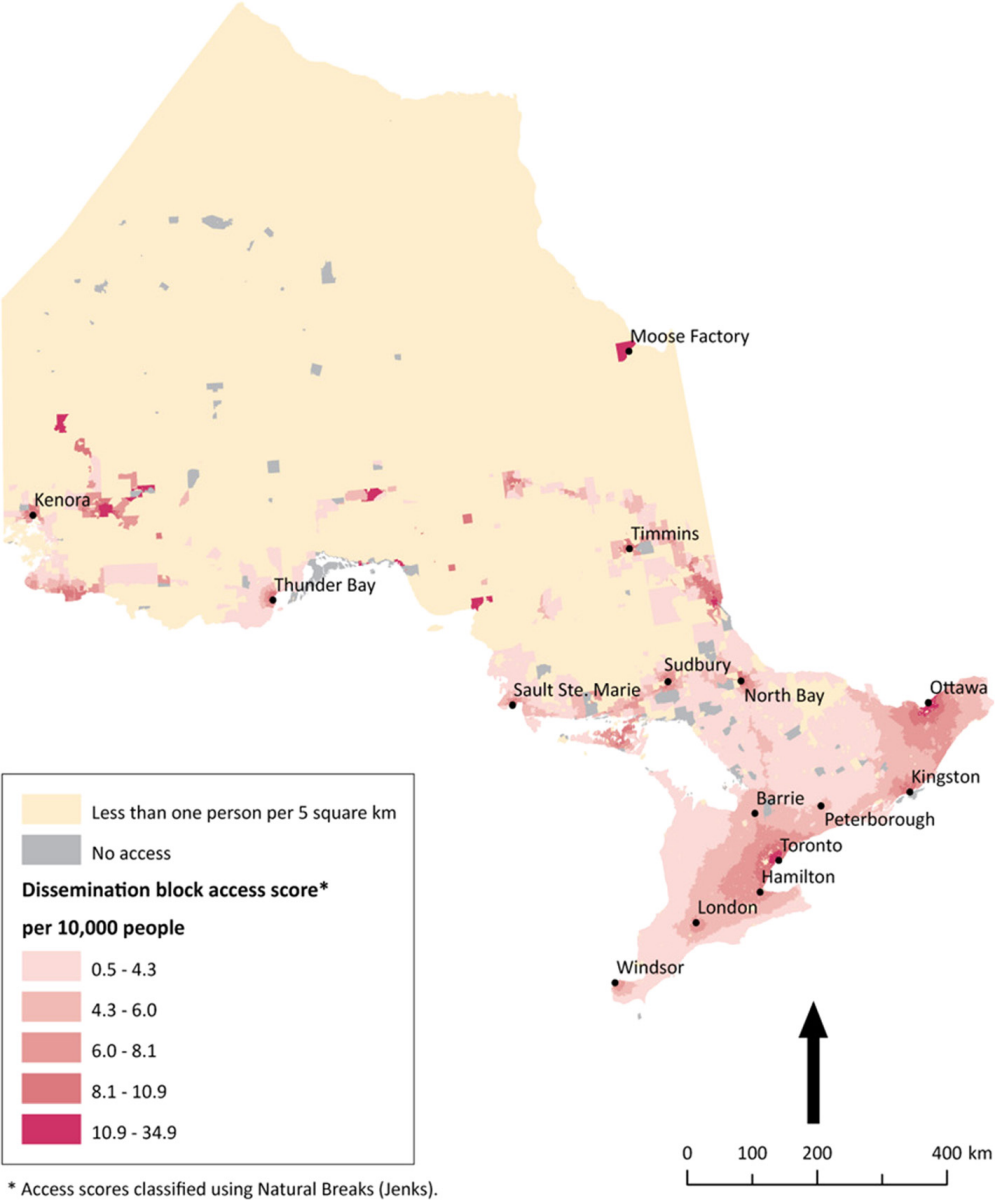
** Potential spatial primary health care physician access in Ontario.** Potential spatial access to primary health care physicians in Ontario. Ontario is Canada’s most populated province. Here there is a distinct north–south pattern of access.

**Figure 5:**
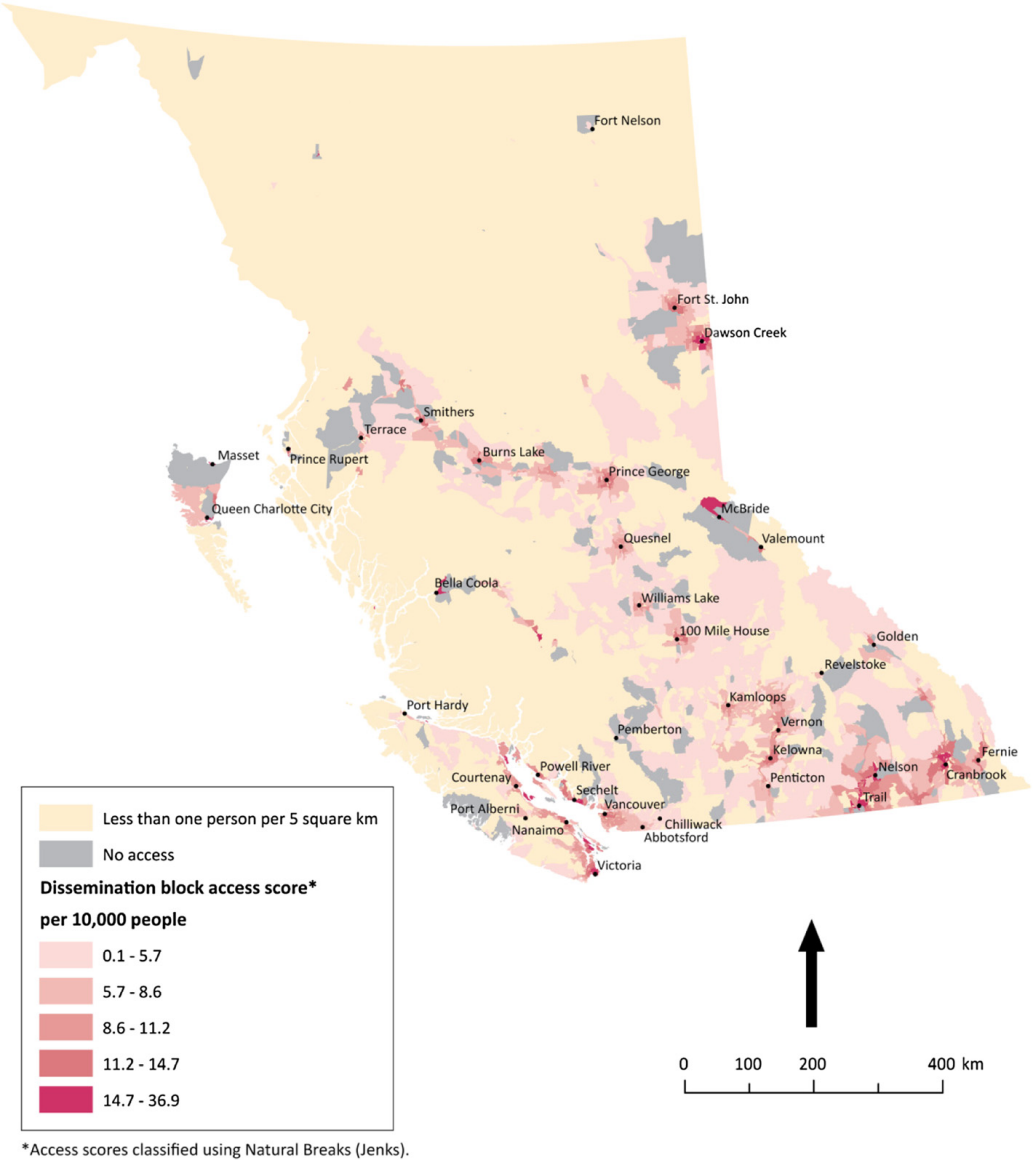
** Potential spatial primary health care physician access in British Columbia.** Potential spatial access to primary health care physician in British Columbia. Inequities of access exist across the province. Here dramatic topography affects both provision of health services and access to them.

For each province or territory, dissemination blocks were mapped and coloured by accessibility score, shown in Figures 2, 3, 4, 5 below. The Jenks Natural Breaks method was used to create access scores using 5 categories, where score distribution does not follow a normal distribution. Dissemination blocks with no access are grey, while low population density areas are cream-coloured. Only dissemination blocks farther than two hours by road or on islands with no road access to a PHC physician should have no access. However, some grey dissemination blocks on the figures do not fit this description. They have not been assigned an access score because they are larger than 5 km^2^ and their centroid is farther than 2.5 km from a road. Two and a half kilometers was chosen as the maximum dissemination block centroid distance from a road to avoid large travel time inaccuracies. For dissemination blocks larger than 5 km^2^, the centroid approximates the population location much less accurately. If the dissemination block is flanked by roads on all sides, the resulting travel time can vary a great deal depending on which road the centroid is closest to. The scores for accessibility in each case show a linear gradient. Interpretation is therefore arithmetic.

Shown in Figure 2, Newfoundland and Labrador is an example of a low population province with residents distributed over thousands of square kilometers. The relatively urban region of the Avalon Peninsula – which houses the capital city of St. John’s – is best served by PHC physicians. However, the surrounding communities do not enjoy this same level of access. Even mid-sized cities, such as Stephenville and Cornerbrook, have generally low potential spatial access scores. The interior of the province has a low population and generally little potential spatial access to PHC physicians. These access patterns are intensified as one moves north. In Labrador (the area attached to the mainland), access to PHC physicians is uniformly minimal. Labrador is typical of arctic and sub-arctic areas, with vast spaces unoccupied. Despite this, two of Labrador’s three cities shown in Figure 2 have no potential spatial access to PHC physicians.

Figure 3 illustrates potential spatial access to PHC physicians in the Nunavut, Canada’s newest territory. Here, potential spatial access is binary. Everyone in the territory is either within 10 minutes of a PHC physician or is completely without access. As such, only two population hubs dominate PHC provision for the expansive territory. There are no roads between these hubs, so while all residents of Iqaluit and Rankin Inlet are within 10 minutes of PHC physicians, the remaining 71% of Nunavut’s inhabitants have no road access to PHC physician clinics.

In Ontario, Figure 4 reveals two distinct patterns of potential spatial access to PHC physicians: the urban south and the rural north. In the highly populated urban south there is reliance on the large population centres in the southeast for provision of care to surrounding areas in the southwest. In southwest Ontario, like in the north, distance is a factor for accessing PHC physicians. Meanwhile, in the province’s north, a familiar Canadian pattern of poor potential spatial access throughout is revealed, with the exception of key small towns that act as centres of health care provision for their more rural and remote hinterlands, such as Moose Factory. Unlike in Nunavut, where much of the expanse that has no access is extremely lightly populated, there are population groupings in Ontario’s north that have no local or regional potential spatial access to PHC physicians. This is particularly true of the northwest.

In the province of British Columbia, topography appears to be influential in determining population patterns and thus patterns of access to PHC physicians. Much of the province is mountainous, with settlement often confined to fertile valleys. As a result, Figure 5 has large (grey) areas with no apparent potential spatial access. Similar to Ontario, the pattern of access for this province shows grouping around the heavily populated areas in the south and minimal potential spatial access to PHC physicians elsewhere. There is not a consistent gradient of access within the most populated areas of British Columbia, as there is across southeast and southwest Ontario. British Columbia’s topography is heavily responsible for this, in that there are few regions in the province that are continuously populated due to the presence of mountains.

Finally, the province of Nova Scotia, illustrated in 6, is an example of the positive effect of an extensive, dense local road network and continuous population on potential spatial access to PHC physicians. Unlike any of the other provinces and territories run in this analysis, Nova Scotia enjoys relatively uniform access. Those few areas that do have ‘no access’ because they are not within 120 minutes of a PHC physician are relatively close to areas ‘with access’ when compared to the proximity of many ‘no access’ communities to ‘with access’ communities in Nunavut, coastal Newfoundland and Labrador, northern British Columbia, and Northern Ontario.

**Figure 6:**
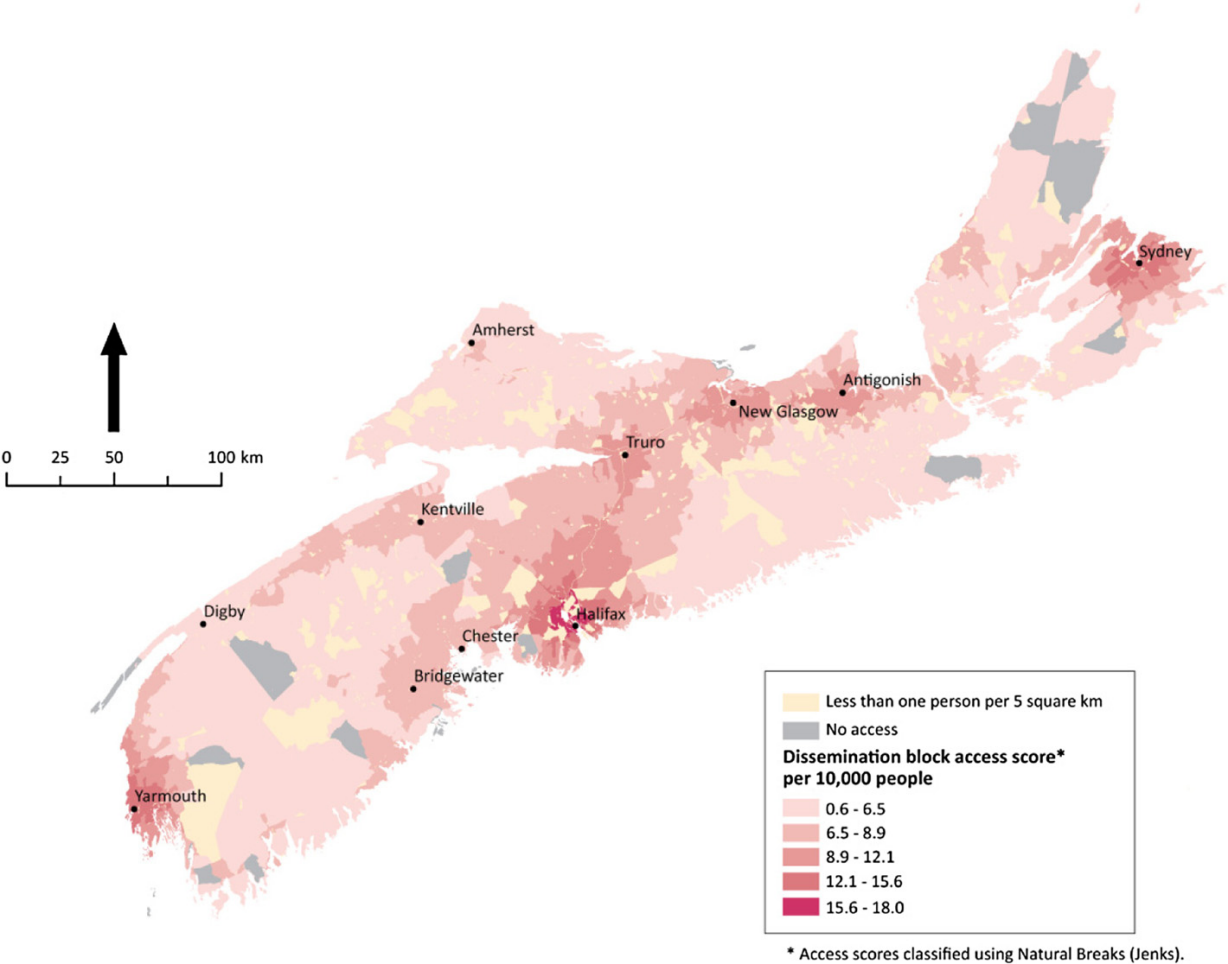
** Potential spatial primary health care physician access in Nova Scotia.** Potential spatial access to primary health care physicians in Nova Scotia. The modified gravity model results show relatively uniform access across the small and lightly populated province.

## Discussion

Based upon the results shared above, distinct spatial patterns emerge regarding potential spatial access to PHC physicians in the five selected Canadian provinces and territories run in this analysis. One is a very clear north–south pattern. Consistent across each of the five provinces and territories is that potential spatial access is greater in southern regions, with those provinces and territories that have vast northern expanses displaying this pattern quite prominently. This is consistent with population distribution in Canada as a whole, where an overwhelming majority of the population lives within 200 kilometres of the country’s southern border [40]. This north–south divide is perhaps most vivid in Ontario, where the southern corridor between Windsor and Ottawa enjoys a strong gradient of access with almost no community falling outside of 120 minutes of drive time to a PHC physician, while the remainder of the province experiences highly varied, but mostly limited, potential spatial access. Another clear pattern emerges at the sub-provincial/territorial level. While gradients of potential spatial access exist within urban and peri-urban centres - such as in southern Ontario, throughout Nova Scotia, and in the southern interior of British Columbia - access is fairly binary outside of these densely-to-moderately populated areas. This is consistent with historical settlement patterns in Canada, with rural and remote townships being spread across vast distances [41].

An important question is: *why* do the distinct spatial patterns regarding potential spatial access to PHC physicians that were characterized above exist? While topography and historical settlement patterns that have shaped current population distribution are certainly relevant, as noted above, the spatial patterns cannot be attributed solely to them. Health services decision-making is another important explanatory factor. In Canada, the majority of health care funding is transferred from the federal government to the provinces and territories, and most of these governments have devolved the main responsibilities for decision-making to the regional level [42]. What this means is that decisions regarding health care priorities and expenditures vary greatly across the country. A recent study that tracked the evolution of a particular type of PHC service in Canada confirmed the existence of this variance, and also found that foundational health policies and funding structures (e.g., the Canada health act), service structures and planning (e.g., focus on service centralization), and health system decisions (e.g., regionalizing decision-making) collectively work to facilitate or impose limitations on service provision [43]. Although these system factors are likely to play a role in shaping the spatial patterns identified through running the modified gravity model, this role is presently uncertain. Addressing this knowledge gap would require consultation with decision-makers whose positions relate to resource allocation, system design, and other central functions of PHC. This is an avenue for future research, both in terms of uncovering the role of administrative decision-making in shaping potential spatial access to PHC physicians and also in gaining guidance on the factors that should be considered when interpreting the findings of the modified gravity model.

The broad patterns of potential spatial access to PHC physicians shown in the results of this analysis are consistent with the findings of others’ research, particularly in relation to the north–south divide in service provision and challenges of affording adequate access in rural and remote regions [29,44-46]. This is an important outcome, because a significant disjuncture between the findings of the modified gravity model and other spatial analyses of access to health services in Canada would suggest a flaw in the model. At the same time, our modification to the gravity model through the inclusion of road network travel time enhances the detail and accuracy of its findings, thus rendering this model not only innovative but also an important contribution to the health services access literature [34]. As health policy and decision-makers continue to invest millions of dollars each year into ‘renewing’ Canada’s PHC system [5,47], it is imperative that researchers provide them with the tools and data they need to make informed decisions [48]. The modified gravity model and results of the analysis presented in this article can serve towards addressing this informational need.

### Limitations

Our approach to determining potential spatial access to PHC physicians does not take a number of factors into consideration. One is that traveling clinics, which are used in many rural and northern areas, are not factored into access calculations. Our road network data has inherent limitations as it does not take into account airborne, water, or snowmobile access to PHC. In a number of rural Canadian communities, especially in the north, snowmobiles, boats, and float planes are routinely used to travel to centres for supplies as well as health care. Perhaps more relevant is that our use of road network travel time does not take into consideration many ‘real-world’ diving conditions such as traffic, weather, and construction. At particular times of the year these factors are likely to have an impact on actual drive time that is not reflected in our theoretical model. Another important limitation pertains to our use of 120 minutes as an indicator of reasonable access. Some people may find this drive to be prohibitive while others may consider drives longer than this to be reasonable.

A notable limitation of our approach is that we have not allowed for the possibility that potential spatial access may extend beyond the border of a particular province or territory, wherein we have treated each province or territory as a bounded unit. Because of the portability clause of the Canada Health Act, Canadians are able to access medical care through the public system outside of their home province or territory. While this clause is most relevant to those who are travelling or in other provinces or territories for short-term stays, it is possible that those living close to the border might seek a regular PHC provider in a proximal town or city that is situated in another province or territory. As the most populated areas of the provinces and territories included in this analysis are not in border areas, however, this practice is not likely to have a significant impact on assessing potential spatial access.

The modified gravity model uses an underlying linear travel impedance assumption. Recent research has, however, suggested that non-linear functions may be appropriate in some instances. For instance, the two-step floating catchment method (a modified gravity model) has been employed in instances where distinct differences or thresholds exist between what is considered accessible and what is deemed inaccessible [49]. These ‘cut-offs’ must be determined in most instances by surveys and thus require the support of place-specific qualitative research [50]. A simpler approach may be to apply weights to different travel times categories, a method employed by Luo and Qi in 2009 [51]. In the case of the current analysis, the scale of the analysis (i.e., entire provinces) and lack of shared understanding of what qualifies as an acceptable travel time to PHC physicians across urban and rural jurisdictions justified the use of a linear travel impedance function.

In an earlier article discussing the gravity model used in the present analysis we offer a lengthy discussion of its limitations [34]. Most relevant to the current article is that the gravity model itself offers a categorical result, which may be considered limiting, and other methods of determining access abound [34]. We fall back on the axiom: all models are wrong; some are useful [52]. In this case, the model offers detailed surveillance of the landscape of potential spatial access to PHC physicians across a vast expanse – hitherto missing from the health services literature.

### Guidance for future applications of modified gravity model

In the introduction we stated that one of the objectives of this article is to provide guidance regarding how findings of the modified gravity model should be interpreted in other analyses. We followed a number of principles in interpreting the results that can be thought of as guidelines for others. First, we did not focus on outliers in the images generated by running the model – which were often numerically insignificant. Rather, we looked for broad patterns. In the case of the present analysis, this strategy was appropriate given the size of Canada. In other applications of the modified gravity model to sizeable geographic areas, this strategy is likely to be applicable. Second, we took into account population density when interpreting the results. This was most important to do when interpreting patterns. We observed areas of low population having very scattered, if any at all, potential spatial access to PHC physicians while areas of high population often having a gradient of access. Third, we attempted to gain an understanding of the local or regional context in order to interpret trends and explain the findings that were observed. It was this process that led us to consider historical population settlement patterns and topography, and to question the role of health service decision-making in shaping the trends that were found. Developing this understanding was also vital to articulating limits of the analysis, as reviewing local context led us to become aware of how in some communities there is standard reliance on non-road-based modes of transportation to access PHC physician clinics. All of which is to say that the model itself is most useful when augmented by qualitative interpretation.

## Conclusions

In this article we examined the potential spatial access to PHC physicians afforded to residents of five Canadian provinces and territories. We did this through running a modified gravity model in GIS that considers road network travel time as one of its variables. The modified model was tested and introduced elsewhere [34], and the current analysis serves as the first opportunity to run it in order to understand how to interpret its results. In interpreting the findings of the GIS outputs, two spatial patterns emerged across the five provinces: a north–south pattern of access and a binary pattern with little gradation. Given these patterns, future GIS studies of potential spatial access to PHC in Canada could usefully experiment with the use of different travel time thresholds for urban and rural residents, as has been done elsewhere [49-51]. Based on having had the opportunity to run the model and interpret its results for this analysis, we offered three recommendations for interpreting the results future applications of the modified gravity model: (1) do not focus primarily on outliers; (2) account for population density; and (3) gain an understanding of the local/regional context. Future applications of the model are needed in order to refine these recommendations on interpreting its results.

PHC is an important aspect of health service delivery within Canada’s public health care system [2]. The front-line care provided by PHC physicians and others involved in the delivery of PHC is vital as existing research has demonstrated that having access to PHC enhances various aspects of health status [11-14]. Because of this, it is important that studies are undertaken that can help administrators, policy-makers, researchers, and others with characterizing the state of access to PHC, including potential spatial access. We encourage further research to be done using GIS in order to offer new, spatial perspectives on issues of access to health services given the increased recognition that the place-based nature of health services can benefit from the use of the capabilities of GIS to enhance the role that visualization and evidence play in decision-making [53]. Such research could involve examining different access perspectives, such as realized spatial access. Alternatively, it could still use a potential spatial access framework while, unlike the current analysis, incorporating spatial dimensions of the demand side such as population characteristics linked to health status that may impact service need and utilization.

## Abbreviations

GIS: Geographic information systems; ODCM: Origin Destination Cost Matrix; PHC: Primary health care.

## Competing interests

The authors declared that they have no competing interest.

## Authors’ contributions

VAC and NS each took primary responsibility for drafting 2 main sections of the manuscript. VAC and NS both edited the manuscript throughout and approved the submission version. VAC took overall responsibility for manuscript style, formatting, and submission. NS originally designed the modified gravity model. VAC and NS worked together to interpret the results of the model for this analysis.

## Pre-publication history

The pre-publication history for this paper can be accessed here:

http://www.biomedcentral.com/1472-6963/12/230/prepub
